# Advances in Organic Small Molecule-Based Fluorescent Probes for Precision Detection of Liver Diseases: A Perspective on Emerging Trends and Challenges

**DOI:** 10.1021/jacs.4c17092

**Published:** 2025-03-04

**Authors:** Luling Wu, Zilu Li, Kun Wang, Robin R. Groleau, Xiaodi Rong, Xueting Liu, Caiyun Liu, Simon E. Lewis, Baocun Zhu, Tony D. James

**Affiliations:** † School of Water Conservancy and Environment, University of Jinan, Jinan 250022, China; ‡ Department of Chemistry, University of Bath, Bath BA2 7AY, U.K.; § School of Chemistry and Chemical Engineering, Henan Normal University, Xinxiang 453007, People’s Republic of China; ∥ Department of Life Sciences, University of Bath, Bath BA2 7AY, U.K.

## Abstract

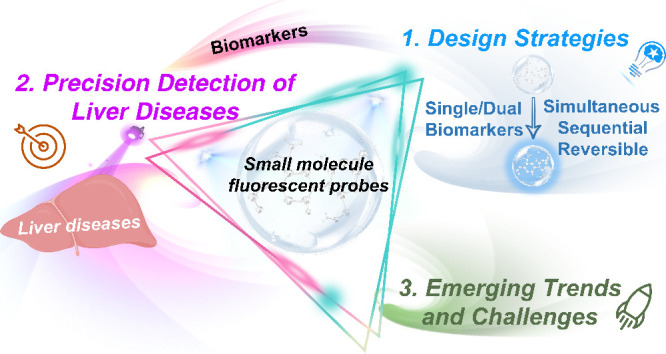

Liver disease poses a significant challenge to global health, and its early diagnosis is crucial for improving treatment outcomes and patient prognosis. Since fluctuation of key biomarkers during the onset and progression of liver diseases can directly reflect liver health and normal/abnormal function, biomarker-based assays are vital tools for the early detection of liver disease. In this context, small molecule fluorescent probes have undeniably emerged as indispensable tools for diagnosis and analysis, with an ever-growing number of small molecule-based fluorescent probes being developed over recent years, with the sole aim of monitoring relevant biomarkers of liver disease. This perspective will focus on the development and application of probes developed primarily over the last 10 years for diagnosing a range liver disease-related processes. It will outline the foundational design strategies for developing promising probes, their optical response to key biomarkers, and how they have been demonstrated in proof-of-concept imaging applications. Current challenges and new developments in the field will be discussed, with the aim of providing insights and highlighting opportunities in the field.

## 1 Introduction

The liver serves as a central hub for a variety of physiological processes, contributing centrally to aspects of macronutrient and xenobiotic metabolism, immune system regulation, endocrine control, and lipid and cholesterol biosynthesis, among others.^1^ It is also, notably, the largest internal organ in the human body, clearly warranting attention and careful understanding, with liver damage or dysregulation readily triggering liver diseases with systemic impacts.^2^ Unfortunately, the incidence of liver disease has recently risen, accounting for an estimated 2 million annual deaths globally (4% of all deaths, which is equivalent to 1 out of every 25 deaths worldwide), as well as broad societal impact associated with such morbidity and mortality. Liver disease and injury includes conditions such as drug-/chemical-induced liver injury (D/CILI), hepatic ischemia-reperfusion injury (HIRI), hepatitis, nonalcoholic fatty liver disease (NAFLD), liver fibrosis (LF), liver cirrhosis (LC) and hepatocellular carcinoma (HCC).^3^

Enhancing patient survival rates and outcomes is ultimately predicated on earlier detection and intervention. One avenue for this is the improvement of biomarker-based detection technologies, of the type discussed throughout this review, which can provide critical insights into early stages of disease. It is important to note that liver disease is a broad umbrella term, encompassing a wide range of conditions, each of which has distinct stages, which are regulated by multiple biomarkers,^4^ with the added inherent complication that biomarker levels and are not static, and fluctuate over time as the diseases progress.^5^ Additionally, the development and progression of many liver diseases is closely linked to changes in the structure and function of organelles, further complicating accurate early detection and diagnosis.^6^ The development of multiparametric and multifunctional diagnostic technologies is therefore paramount, with a focus on simultaneous detection of multiple biomarkers or parameters related to liver disease in order to more effectively address the complexity and diagnostic challenge associated with these conditions.

**Figure 1:**
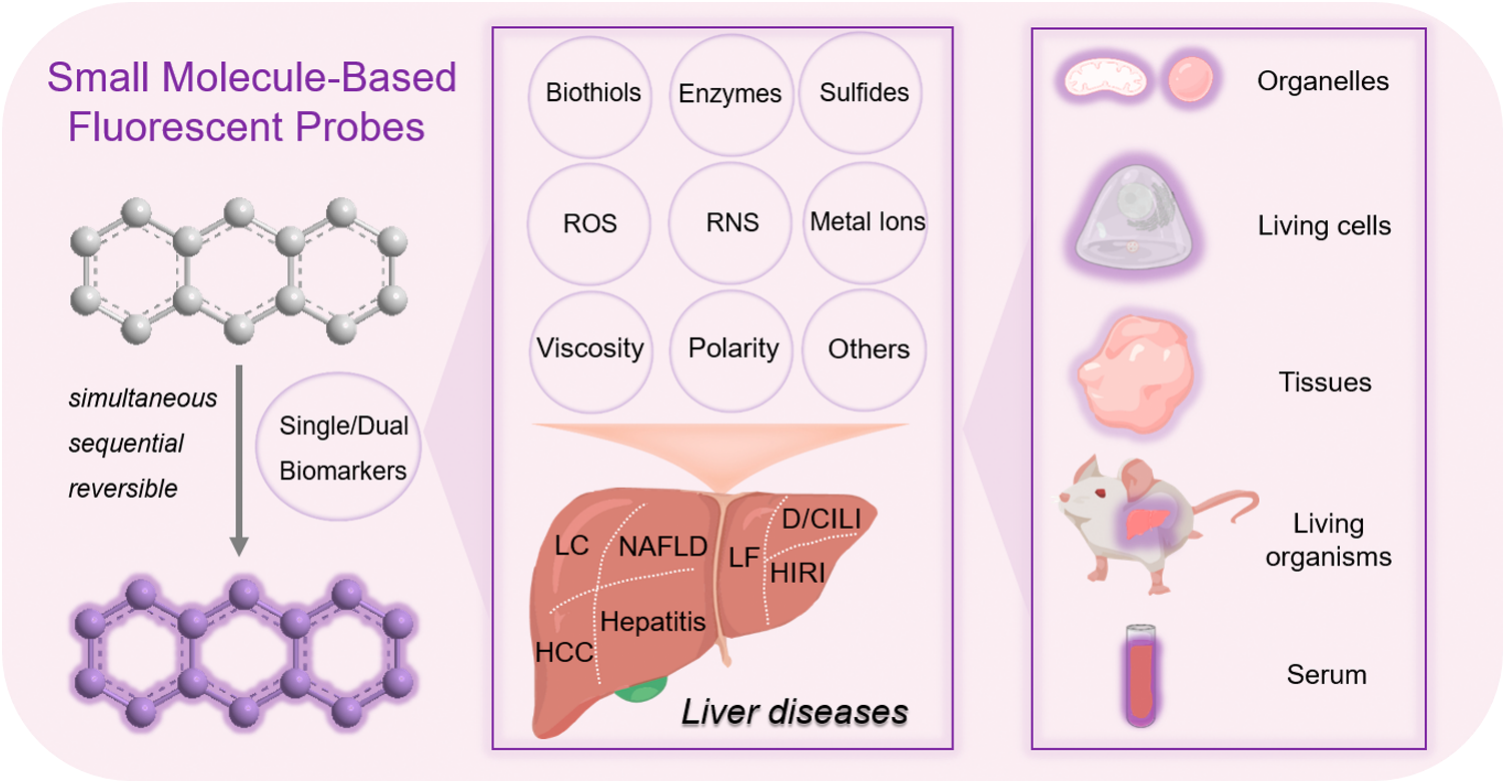
Biomarkers associated with liver diseases and the use of small molecule based fluorescent probes for the detection of liver disease at multiple levels, including organelles, cells, tissues and living organisms.

Other sensing technologies, such as quantum dots and chemiluminescence imaging, aim to address many of these challenges. However, many chemiluminescent substrates exhibit weak luminescent intensity under physiological conditions. Additionally, quantum dots often face restrictions in certain applications due to their potential toxicity.^7^ As such, thanks to their characteristic small size, good permeability, high sensitivity, excellent and tunable selectivity, and real-time detection capabilities,^8^ small molecule-based fluorescent probes have emerged as a popular tool for liver disease diagnosis and imaging through the detection of representative biomarkers.^9,10^ To meet the challenges outlined above, the design of small molecule probes has gradually evolved toward the development of multifunctional and highly integrated multiparametric probes (Figure 1). Both single- and dual-responsive probes exist, and both types will be outlined throughout this perspective, though dual-responsive systems are becoming more prevalent. Both types of probes can be substantially modified and tuned to meet very specific imaging requirements in order to enable their use for a range of diseases and facilitates imaging at multiple levels, from organelles to entire living organisms (Figure 1). Thanks to this flexibility, small molecule fluorescent probes are capable of simultaneously, sequentially, dynamically, and sometimes reversibly monitoring multiple key biomarkers during complex physiological and pathological processes, providing critical information regarding the location, extent, and nature of lesions.

This perspective aims to outline key aspects in the development and application of small molecule-based fluorescent probes for the detection of various types of liver disease, presenting general design strategies and highlighting potential developmental opportunities. The focus is placed on highly representative examples of probes specifically developed for the imaging of various liver diseases. It should be noted that the focus of this perspective is on small molecule-based fluorescent probes, primarily covering research from the last 10 years, and systems based around other technologies such as quantum dots or chemiluminescence are not included. In highlighting key challenges and future opportunities, alongside suggestions for future directions of investigation, we hope to inspire and guide researchers in the development of ever more precise, multifunctional, and multiparametric small molecule-based fluorescent probes for improved detection, study, and diagnosis of liver disease.

## 2 Design Strategies toward Fluorescent Probes for Liver Disease

### 2.1 Single-Responsive Fluorescent Probes

**Figure 2:**
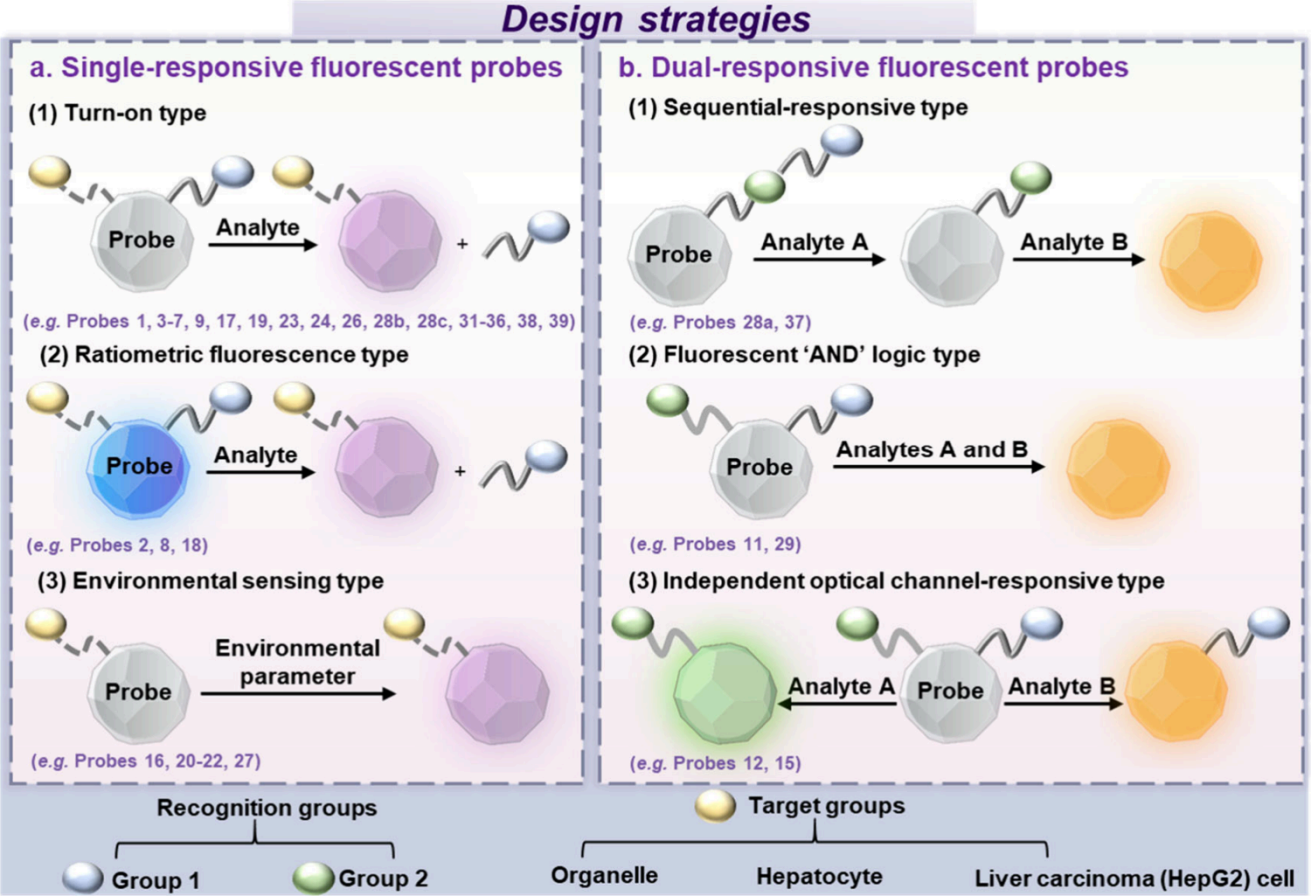
Fluorescent probe design strategies: (a) single-responsive fluorescent probes; (b) dual-responsive fluorescent probes.

Single-responsive fluorescent probes are designed to detect changes in a single analyte, and so the associated fluorescence signal and its fluctuations depends entirely on this single analyte.^11^ The design strategies for single-responsive probes will be outlined in this perspective. (1) “Turn-on type” (Figure 2a1), where the probe interacts/chemically reacts with a specific biomarker to trigger changes in the fluorescence signal. This is the simplest approach to probe design. (2) “Ratiometric fluorescence type” (Figure 2a2), where multiple wavelengths are monitored for a given probe, with the ratio between them indicating analyte concentration. This approach mitigates interference from extraneous factors, thereby elevating the precision and reliability of detection. (3) “Environmental sensing type” (Figure 2a3), wherein probes can reversibly change their fluorescence characteristics by sensing changes in environmental factors (such as pH value, viscosity, polarity) rather than specific analytes.^12^

These probes can undergo chemical modifications to confer organelle/hepatocyte/liver carcinoma (HepG2) cell-targeting capabilities. Most common is organelle-targeting, where the most commonly employed strategy is molecular tagging, where a targeting group is introduced to enable localization of the probe at the desired site.^13,14^ For instance, hepatocytes/liver carcinoma (HepG2) cells can be targeted via specific receptors. By leveraging the specificity of these receptors, it is possible to design fluorescent probes that can specifically bind to them, and therefore selectively target hepatocytes. Thus, the core structure of these probes typically comprises of three components: an organelle/hepatocyte/liver carcinoma (HepG2) cell-targeting group; a fluorophore; and a recognition unit, all covalently linked to form a single probe.

### 2.2 Dual-Responsive Fluorescent Probes

The close relationship between specific biomolecules means that it can be beneficial to monitor more than one species concurrently, especially in the case of initiation and progression of liver disease, where the specific balance of key biomarkers plays a crucial role. This can be achieved with dual-responsive probes, which typically exhibit greater specificity for the early screening of liver diseases when compared to single biomarker detection-based probes. These dual-responsive systems are also valuable tools for the study of complex underlying disease mechanisms, inherently monitoring the interplay between multiple interconnected biomarkers.

Simply put, dual-responsive probes respond to two biomarkers, either requiring both in order to elicit a response, or exhibiting a differential response dependent on which of the two has been detected, as determined by the selected design strategy.^15−17^ The following approaches will be outlined here: (1) “Sequential-responsive type” requires both biomarkers to activate the probe in a specific order to elicit a fluorescence response. In this case, if one of the biomarkers is present only in trace amounts in the diseased state, a probe can only provide a low (or no) fluorescence output (Figure 2b1); (2) “Fluorescent “AND” logic type-based” probes act similarly, but no specific order of activation is required (Figure 2b2); (3) “Independent optical channel-responsive type” operate such that each analyte (and combination thereof) elicits a different fluorescent response, which are imaged using separate channels.^18^ The simultaneous imaging of two biomarkers in independent channels not only helps understand the correlation between different biomarkers in a given pathology, but also helps distinguish the specific contributions of different biomarkers, while also improving the accuracy of disease diagnosis (Figure 2b3). These three classes encompass the majority of dual-responsive probes, but there are of course other types of dual-responsive probes that exist, which this perspective will not have the opportunity to cover. As already outlined for single-responsive probes, dual-responsive probes can be modified with a range of targeting groups.

In this perspective, as illustrated in Figure 2, we have grouped each class of probe to help readers appreciate the functional similarity between them. However, for probes **30**, **40**, and **41**, which are nonresponsive fluorescent probes, probes **13**, **14** and **25** which are redox-reversible fluorescent probes, and probe **10** which is a fluorescent/chemiluminescent dual-modal probe, they have not been included in Figure 2, these probes do not fit the simple classifications and will be discussed separately .

## 3 Probes for Liver Disease Imaging

### 3.1 Drug/Chemical-Induced Liver Injury (D/CILI)

**Figure 3:**
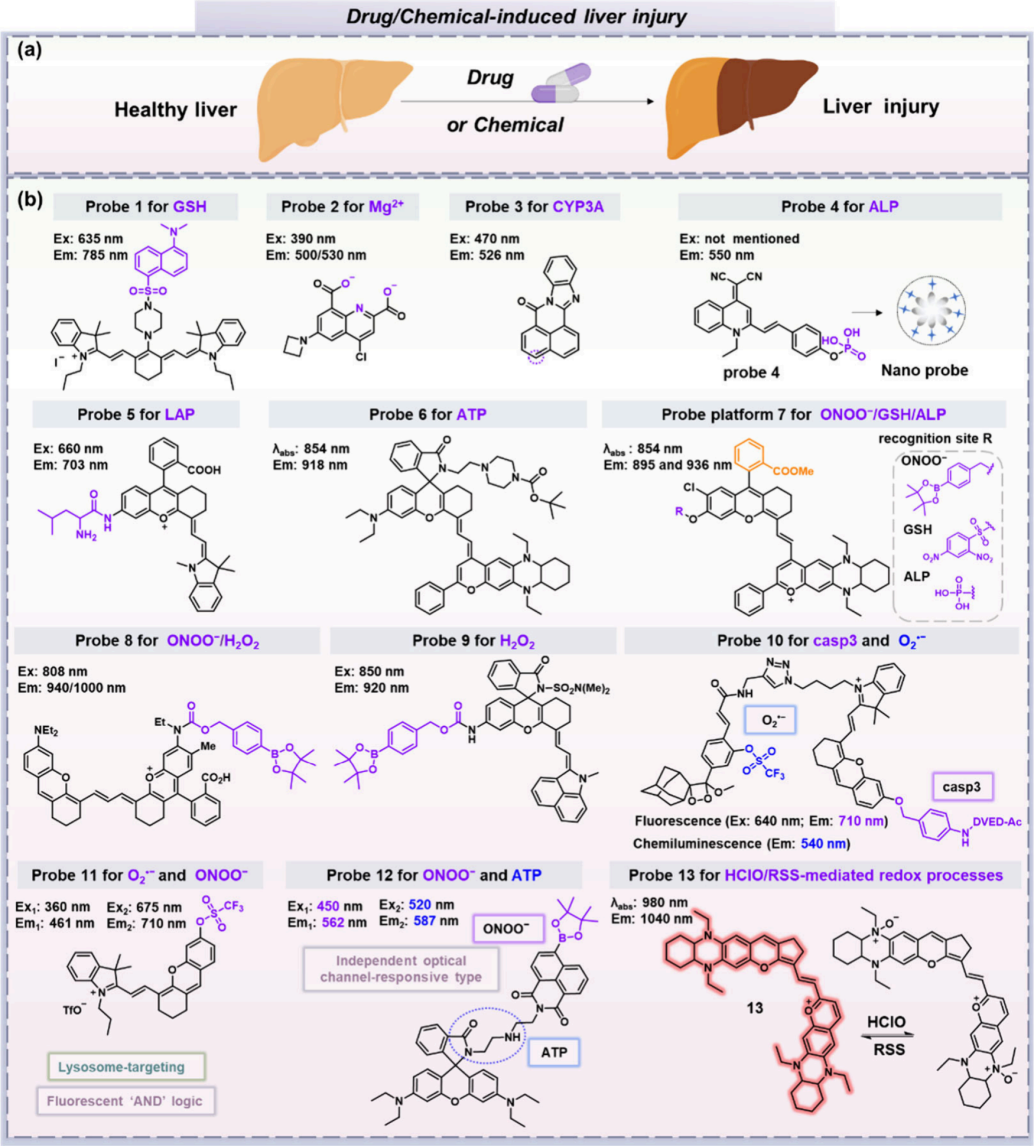
(a) Schematic of how drug overdose or chemical exposure can cause D/CILI. (b) Chemical structures of fluorescent probes **1**–**13** for D/CILI. For the probe structures **1**–**13**, the purple/blue part represents the reactive moiety, the orange part of probe **7** represents the methyl benzoate group. GSH, glutathione; Mg^2+^, magnesium ions; CYP3A, cytochrome P450 3A; ALP, alkaline phosphatase; LAP, leucine aminopeptidase; ATP, adenosine triphosphate; ONOO^–^, peroxynitrite; H_2_O_2_, hydrogen peroxide; O_2_^•–^, superoxide anion; casp3, caspase-3; RSS, reactive sulfur species; HClO, hypochlorous acid.

Acute liver injury (ALI) is characterized by a rapid loss of hepatocyte function, and is often caused by drug-induced liver injury (DILI) and chemical-induced liver injury (CILI) (Figure 3a).^19^ Traditionally, assessing the severity of ALI involves evaluating serum biomarkers, however these blood tests often lack the sensitivity and specificity required for early diagnosis, as well as being influenced by a variety of other diseases, thus they do not always accurately reflect rapid or recent changes in liver function. This illustrates the urgent need for the development of small-molecule fluorescent probes for the detection of biomarkers associated with DILI and CILI, facilitating early detection of ALI (Figure 3b).

Acetaminophen (APAP, paracetamol) is a common cause of DILI, with excessive doses of APAP resulting in the buildup of *N*-acetyl-*p*-benzoquinone imine (NAPQI) to toxic levels. NAPQI is rapidly detoxified by glutathione (GSH), and is ultimately eliminated as the NAPQI-GSH adduct.^20^ In 2014, Ryu, Yoon and colleagues developed a cyanine dye-based fluorescent probe **1**,^21,22^ employing a sulfonamide motif for the selective detection of GSH. On reaction with GSH, the 5-(dimethylamino)naphthalenesulfonamide group is cleaved to cause a turn-on response. Probe **1** was successful in monitoring changes in GSH concentration resulting from APAP-induced liver injury, demonstrating excellent single-responsive detection of GSH over other biological thiols. Other interactions can also be harnessed, as is the case in probe **2**, developed by Martínez-Chantar, Buccella and colleagues in 2023 for the detection of magnesium ion (Mg^2+^) deficiency, which has been linked with LC, alcoholic liver disease, and other ALIs.^23^ Probe **2** was designed around a quinoline bis(carboxylate) motif capable of strong multidentate coordination with Mg^2+^, enabling its detection with good selectivity and sensitivity. In an APAP-induced liver injury model, flow cytometry revealed a decrease in intracellular Mg^2+^ levels.

Enzymes are also readily detected using small molecule-based probes, such as cytochrome P450 3A (CYP3A), alkaline phosphatase (ALP), or leucine aminopeptidase (LAP), all of which play a significant role in the progression of DILI. In 2022, Feng, Ma and colleagues developed a two-photon fluorescent probe **3** for tracking CYP3A activity changes.^24^ This naphthalimide-like probe employs an interesting design strategy which requires hydroxylation of the probe by CYP3A at the highlighted position to turn on fluorescence, requiring neither a linker nor additional synthetic steps to produce a functional small molecule probe. Bypassing the need for liposome production using surfactants, Peng, Yoon and colleagues designed an amphiphilic fluorescent probe **4** capable of self-assembly.^25^ In this case the phosphate unit acts as both the ALP recognition site and a polar headgroup. On activation by ALP, the probe’s fluorescence is restored, and its amphiphilic properties are suppressed. Probe **4** was used to successfully monitor changes in the activity in APAP-induced liver injury. Additionally, this probe could be used to distinguish between healthy tissue and liver cancer tissue thanks to ALP overexpression in abnormal cells. Another key development is the use of near-infrared (NIR) fluorescence motifs which enable better tissue penetration, as demonstrated by probe **5**.^26^ Developed by Yuan and colleagues, this high-fidelity probe was employed for *in vivo* imaging to monitor APAP-induced hepatotoxicity through changes in LAP activity.

Unsurprisingly, adenosine triphosphate (ATP), and particularly its production, can serve as a key indicator of dysregulation. Drug-induced liver damage is known to disrupt ATP synthesis, thus impairing normal liver function. In 2020, Zhang et al. developed a second NIR window (NIR-II, 900–1700 nm) fluorescent probe **6** for detecting ATP.^27^ In its inactivated state, this probe exhibits only weak fluorescence, but upon binding with ATP the fluorescence intensity is switched on. In a mouse model of APAP-induced liver injury, probe **6** was used for high contrast noninvasive imaging of ATP level changes. Building on the foundation of probe **6**, future developments could involve optimizing the structure to enable the detection of additional biomolecules, not only metal ions and ATP,^28^ thereby being flexible and adaptive for the analysis of other pathological conditions.

More versatile probes with modifiable/adaptable alcohol or amino groups have a much wider scope of application, with opportunities for use in the detection of multiple analytes following only simple modifications. For instance, Yuan et al. improved the hemicyanine dye (HD) platform by replacing the indole heterocycle with a 1,4-diethyl-decahydroquinoxaline benzopyran group and adding a methyl benzoate group (the orange part) as a capping group into the HD to develop a NIR-II fluorescent platform **7**.^29^ Utilizing this platform, they reported three activatable NIR-II probes for detecting either peroxynitrite (ONOO^–^), GSH, or ALP depending on the reactive motif installed onto the hydroxyl group. Utilizing the reactive oxygen species (ROS)- and thiol-detecting probes, changes in the redox state of liver tissue during APAP-induced liver injury were readily evaluated. This clearly highlights the versatility and opportunities provided by such highly tunable probes, laying a solid foundation for the future development of specific NIR-II probes for detecting other liver-disease related biomarkers.

Lei et al. and Li et al. both introduced NIR-II fluorescent probes based on a rhodamine scaffold. Lei et al. reported a ratiometric NIR-II fluorescent probe **8** constructed by integrating a rhodamine 6G scaffold with polymethine.^30^ Subsequently, the authors demonstrated the practicality of ROS probe **8** (for detecting H_2_O_2_ and ONOO^–^) by detecting strong ratiometric signals in an APAP-induced liver injury mouse model. Similarly, Li et al. introduced a rhodamine-based hybrid NIR-II dye with an amine substituent **9** for the detection of H_2_O_2_ using dual-modality fluorogenic/photoacoustic (PA) imaging. Examples discussed so far have used APAP to induce liver damage, however trazodone has also been associated with liver injury. As such, Li et al. used probe **9** to evaluate upregulation of H_2_O_2_ in a trazodone-induced liver injury model, confirming its ability to monitor oxidative stress during liver damage beyond classical APAP-induced liver injury in real time.^31^

Looking now at dual-responsive probes, let us consider drug-induced hepatotoxicity (DIH) which is a significant etiology of DILI. In the early stages, oxidative stress and apoptosis are typically upregulated, before the onset of inflammatory responses and eventual liver failure. With this in mind, Pu et al. reported a fluorescent probe **10** that integrates two independent signaling activation pathways: NIR fluorescence and chemiluminescence.^32^ The NIR fluorescence channel was designed to detect caspase-3, a critical protease activated during apoptosis, while the chemiluminescence channel monitored superoxide anion (O_2_^•–^). Experiments confirmed that this probe was capable of detecting DIH prior to observable histological changes, indicating its potential use as a highly sensitive optical reporter for early DIH detection. Beyond this specific application, this type of molecular design strategy can be extended to the dual imaging of other biomarkers by simply modifying the pendent analyte-reactive units.

As a key biomarker of DILI and oxidative stress in general, O_2_^•–^ garners significant attention. In particular, O_2_^•–^ can react with endogenous nitric oxide to produce ONOO^–^, a very reactive nitrogen species. Thus, detection of O_2_^•–^ and ONOO^–^ can provide information on the synergy of these two species in DILI. In this context, Li, Tang, James and colleagues have reported a “fluorescent “AND” logic type” probe **11**. Designed to detect two key oxidative stress biomarkers O_2_^•–^ and ONOO^–^,^33^ probe **11** does not require a specific order of activation to produce the final product’s blue fluorescence. The probe combines NIR fluorescence and two-photon excitation fluorescence techniques, allowing for the detection of changes in O_2_^•–^ and ONOO^–^ in APAP-induced injury models. Building on this, Han, Zhang, Huang, Li, James, Sessler and colleagues aimed to improve this platform by designing a probe capable of independently detecting two analytes in different emission channels. They therefore designed an “independent optical-channel responsive” fluorescent probe **12** for simultaneously monitoring concentration changes of ONOO^–^ and ATP in cells.^34^ In this instance, the boronate ester group acts as the reactive site for ONOO^–^, with a modified rhodamine moiety enabling specific response to ATP. This design approach illustrates the potential for highly customized designs targeting two specific analytes, reinforcing the modularity shown in probes **7**, **8**, and **9** above: by incorporating the appropriate reactive site into the probe scaffold, it is possible to achieve a specific response to a single analyte. Using probe **12**, the authors successfully monitored the increase of ONOO^–^ and concomitant depletion of ATP during APAP-induced hepatotoxicity, demonstrating its potential for practical applications. These two research projects illustrate the power of dual-responsive fluorescent probes (**11** and **12**) for the monitoring of DILI related biomarkers, aiming to enhance the accuracy of diagnostic assessments in DILI.

Carbon tetrachloride (CCl_4_) is a substance which is metabolized by cytochrome P450 2E1 to produce highly reactive species and is widely used to induce CILI in animal models. Based on this, Ren, Yuan and colleagues have devised a reversible NIR-II fluorescent probe **13**, which was based on the trimethine cyanine skeleton.^35^ By incorporating a 1,4-diethyl-decahydroquinoxaline moiety into their molecular architecture, a significant redshift in wavelength and excellent response to hypochlorous acid was obtained. Furthermore, this moiety can be reduced by reactive sulfur species (RSS). As such probe **13** has been successfully utilized to detect alterations in the oxidative microenvironment in models of CCl_4_-induced liver injury/repair processes.

### 3.2 Hepatic Ischemia-Reperfusion Injury (HIRI)

**Figure 4:**
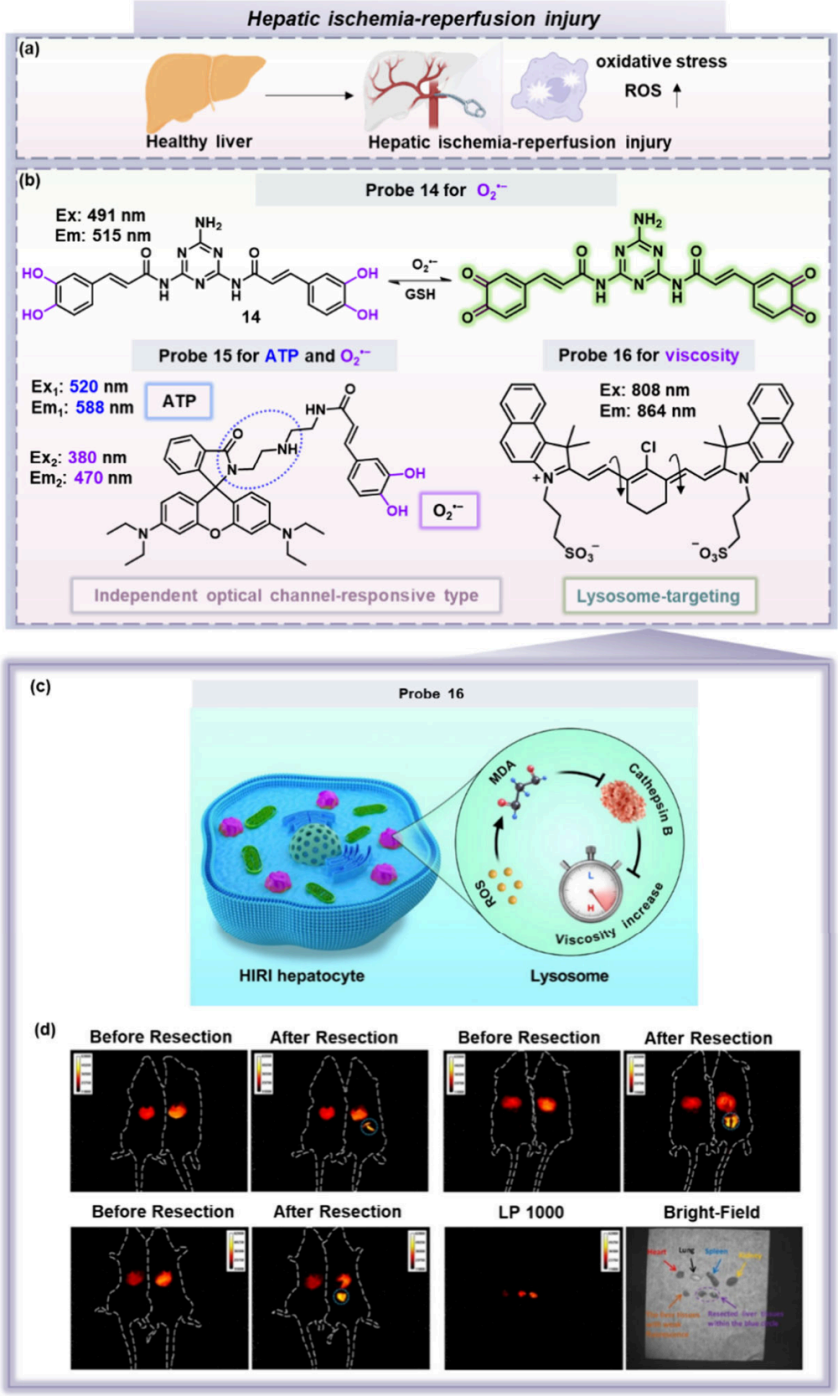
(a) Oxidative stress is one of the important mechanisms of HIRI. (b) Chemical structures of fluorescent probes **14**–**16** in HIRI. For probe structures **14**–**16**, the purple/blue part represents the reactive moiety. (c) Schematic of lysosomal ROS–MDA–Cathepsin B cascade signaling pathway-mediated viscosity change during HIRI, (d) NIR-II fluorescence imaging in liver lesions in HIRI mice using probe **16**. ROS, reactive oxygen species; MDA, malondialdehyde. Image (c) reproduced and (d) adapted with permission from ref (39). Available under CC-BY 4.0 license. Copyright 2022, the authors (J. Liu, W. Zhang, C. Zhou, M. Li, X. Wang, W. Zhang, Z. Liu, L. Wu, T. D. James, P. Li and B. Tang). Published by American Chemical Society.

HIRI is a common pathophysiological phenomenon that typically occurs when blood flow to the liver is temporarily interrupted and then restored, as may happen during liver transplant, partial hepatectomy, or cardiac surgery. Unraveling the molecular mechanisms of HIRI at the molecular level and rapidly and accurately pinpointing early HIRI lesions is crucial for timely treatment and reducing adverse patient prognoses. In the early stages of HIRI, the liver generates a considerable amount of ROS. These highly reactive ROS can induce oxidative stress, leading to hepatocyte damage and cell death. Subsequently, ROS continues to be produced during reperfusion, creating a vicious cycle.^36^ Furthermore, during HIRI, the homeostasis of the microenvironment is markedly disrupted. Therefore, recent research has delved into the pathogenesis of HIRI, focusing on the dysregulation of oxidative stress and alterations in the microenvironment (Figure 4a), contributing significantly to our understanding of this condition.

For oxidative stress, Tang et al. developed a fluorescent probe **14** for monitoring the dynamic changes of O_2_^•–^.^37^ This probe is based on a triaminotriazine framework and it is constructed by covalently attaching two caffeic acid molecules. In the presence of O_2_^•–^, the caffeic acid residues of the probe undergo a transformation from pyrocatechol to *ortho*-benzoquinone, allowing for highly selective detection of O_2_^•–^. Importantly, this transformation is reversible; the addition of GSH can promote a reduction reaction, leading to a decrease in fluorescence intensity (Figure 4b). This reversibility facilitates the dynamic monitoring of O_2_^•–^ levels. Using this probe, a marked increase in O_2_^•–^ levels was observed in mice subjected to HIRI, which demonstrated the critical role of O_2_^•–^ in HIRI. In the future, based on this pioneering work, we believe that research should focus on developing long-wavelength fluorescent probes to enable deeper tissue imaging of HIRI in living organisms.

The progression of HIRI is not only linked to oxidative stress but also often accompanied by disruptions in energy metabolism. ATP, known as the “molecular currency” of energy transfer, is a critical marker in the process of HIRI, with its depletion during ischemia being a significant indicator. Zhang, Wu, James, Li, Tang and colleagues utilized a rhodamine lactam skeleton that was combined with a caffeic acid moiety to develop a dual-color and reversible molecular fluorescent probe **15** for the real-time detection and dynamic imaging of O_2_^•–^ and ATP in HIRI.^38^ Using this probe, synchronous bursts of O_2_^•–^, and depletion of ATP in HIRI, along with a slight increase in ATP during reperfusion was observed. Unlike traditional single-targeting probes, probe **15**’s dual-channel design provides comprehensive insights into oxidative stress and energy status, especially valuable for early stage HIRI detection and intervention. The probe’s two channels (blue for O_2_^•–^ and red for ATP) are separated by a spectroscopic interval of 118 nm, effectively preventing signal crosstalk and enhancing imaging accuracy. This design is essential for effective dual-responsive probes, offering high-precision detection in complex biological environments. Overall, probe **15**’s dual-responsive, reversible strategy demonstrates the potential for real-time dual-biomarker monitoring. Future designs of related probes could explore additional redox-sensitive structures or multiresponse sites to detect a broader range of biomarkers in complex HIRI pathological settings.

Oxidative stress during HIRI leads to alterations in the hepatocyte microenvironment. For instance, it can impair lysosomal degradative functions, resulting in significant changes in lysosomal viscosity. Therefore, lysosomal viscosity is closely related to the development of HIRI. Considering lysosomal viscosity as a key marker for HIRI, Zhang, Wu, James, Li, Tang and colleagues integrated the structural advantages of indocyanine green (ICG) and IR-783 to develop a NIR-II fluorescent probe **16**.^39^ ICG exhibits its fluorescence primarily in the NIR window, however, it is prone to photodegradation. In contrast, IR-783 has higher photostability, which is attributed to the presence of a rigid cyclohexene moiety at its central position. Therefore, by adding multiple aromatic rings and a cyclohexenyl ring, the emission wavelength and photostability of probe **16** could both be effectively improved. Probe **16** successfully achieved real-time imaging of lysosomal viscosity changes in hepatocytes and mouse models. The study revealed a ROS–malondialdehyde (MDA)–cathepsin B signaling pathway in HIRI and confirmed that lysosomal viscosity is an ideal biomarker for precise imaging of HIRI (Figure 4c, d). Building on this research, future approaches should develop probes to investigate other signaling pathways and mechanisms associated with the onset and progression of HIRI.

### 3.3 Hepatitis

**Figure 5:**
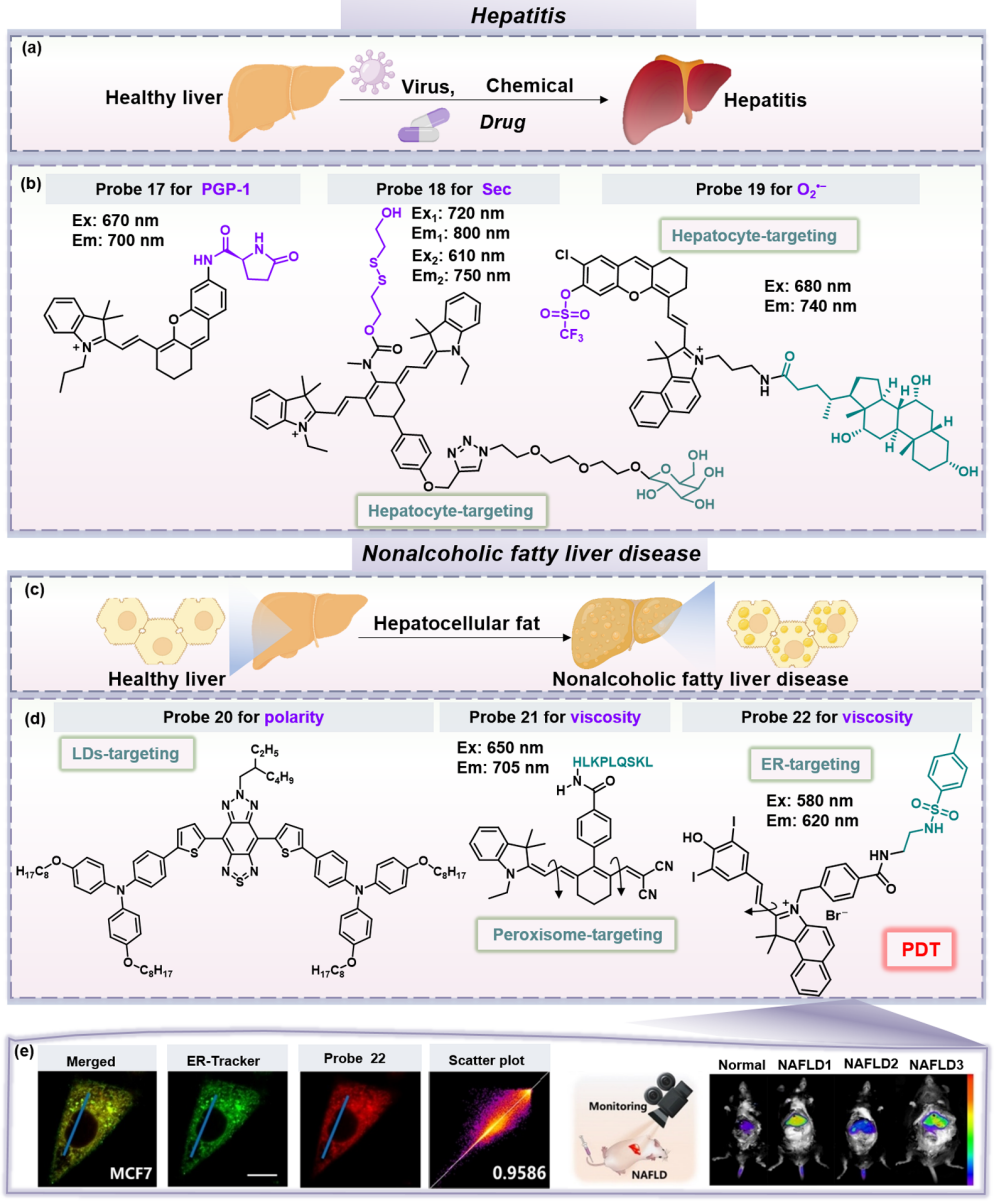
(a) Hepatitis can be triggered by various factors. (b) Chemical structures of fluorescent probes **17**–**19** for hepatitis. (c) NAFLD is characterized by the abnormal accumulation of fat in the liver. (d) Chemical structures of fluorescent probes **20**–**22** for NAFLD. Probe structures **17**–**22**, the purple part represents the reactive moiety, and the green part represents the organelle/hepatocyte-targeting group. (e) The colocalization imaging between probe **22** and ER-Tracker Green in MCF7 cells, and in *vivo* liver imaging of normal and NAFLD mice after intravenous injection of probe **22**. PGP-1, pyroglutamate aminopeptidase 1; Sec, selenocysteine; LDs, lipid droplets; ER, endoplasmic reticulum; PDT, photodynamic therapy. Image (e) adapted with permission from ref (55). Copyright 2024 Wiley-VCH GmbH.

Hepatitis, an inflammatory condition of the liver, can be triggered by various factors such as viral infections, chemical poisons and drugs (Figure 5a).^40^ Due to the diversity and complexity of the pathogenic mechanisms of hepatitis,^41^ a comprehensive understanding of the disease’s etiology requires further research. Establishing animal models is an effective approach for investigating this disease. Currently, there are various models for inducing acute hepatitis, including those that use pharmacological agents to simulate the disease’s pathogenic mechanisms. To date, researchers have developed a series of small molecular fluorescent probes designed to detect bioactive substances involved in hepatitis models.^42,43^ These probes provide valuable visual tools for the early diagnosis and treatment of hepatitis.

For instance, a mouse model of hepatitis can be established through the combined administration of lipopolysaccharides/d-galactosamine, which effectively mimics the mechanisms underlying inflammation.^44^ The relationship between pyroglutamate aminopeptidase 1 (PGP-1) and hepatitis has garnered increasing attention. Wu et al. designed a novel NIR fluorescent probe **17** by incorporating l-pyroglutamic acid into the framework of the HD fluorophore (Figure 5b),^45^ the addition of PGP-1 releases the fluorophore with restoration of the fluorescence signals. Subsequently, researchers observed that the expression of PGP-1 was upregulated in the lipopolysaccharides/d-galactosamine-treated liver inflammation mouse group. This work provides significant insights into the mechanistic role of PGP-1 in liver inflammation, and lays a foundation for the development of therapeutic targets for hepatitis.

Additionally, a high dose of CCl_4_ can induce ALI, serving as another model for acute hepatitis. Selenocysteine (Sec) serves as a crucial active site residue in various antioxidant selenoproteins, and intracellular levels of free Sec are associated with inflammatory diseases such as acute hepatitis. However, due to its high reactivity and instability, the detection of Sec in live cells and *in vivo* remains challenging. Yu, Chen and colleagues developed a ratiometric near-infrared fluorescent probe **18** for the qualitative and quantitative measurement of Sec in live cells and *in vivo*.^46^ The probe consists of a heptamethine cyanine fluorophore, a response unit based on bis(2-hydroxyethyl) disulfide, and a hepatocyte-targeting moiety (d-galactose), which enhances its hepatocyte-targeting capability (Figure 5b). The quantitative analysis of Sec fluctuations in cellular and mouse models was achieved using ratiometric fluorescence signals from the probe. Experimental results indicate that Sec plays a significant antioxidant and anti-inflammatory role during inflammatory processes, and the intracellular levels of Sec are closely correlated with the severity of liver inflammation.

Conditions like autoimmune hepatitis can lead to an increased production of O_2_^•–^ that surpass the body’s detoxification capacity, culminating in oxidative stress. Yuan, Gao, Su and colleagues reported a novel NIR fluorescence/PA dual-modality O_2_^•–^ probe **19** (Figure 5b).^47^ Similar to probe **18**, probe **19** features hepatocyte-targeting performance thanks to the cholic acid motif. Overall, probes **17**–**19** represent progress toward hepatitis imaging, emphasizing the design of probes with hepatocyte-specificity and inflammation-response, which can improve diagnostic accuracy. Future work could build on this research, by enhancing multibiomarker detection capabilities, targeting specificity, and biocompatibility, which will enable the more comprehensive and accurate diagnosis of hepatitis.

### 3.4 Nonalcoholic Fatty Liver Disease (NAFLD)

NAFLD is a liver condition characterized by the abnormal accumulation of fat in the liver, unrelated to alcohol consumption (Figure 5c). If not diagnosed and treated promptly, NAFLD may progress to liver inflammation, LF, and even LC or HCC.^48−50^ The onset of NAFLD is closely associated with multiple factors, among which oxidative stress is considered a key element. Additionally, the progression of NAFLD is intricately linked to intracellular lipid metabolism. Abnormal lipid metabolism can lead to lipid accumulation and the formation of lipid droplets (LDs), accompanied by changes in the cellular microenvironment, such as polarity and viscosity. Recently, researchers have developed various types of fluorescent probes based on different NAFLD-related biomarkers.

The abnormal accumulation of LDs in hepatocytes is a prominent feature of NAFLD. Therefore, *in situ* monitoring of LDs change in the liver can provide a direct assessment of fat accumulation, thereby aiding in the diagnosis of NAFLD. Numerous fluorescent probes have been developed for imaging LDs in NAFLD.^51,52^ For example, Li, Qin, Wu and colleagues reported a novel NIR polarity-responsive LD-targeted fluorescent probe **20** for monitoring the progression of NAFLD in live mouse models (Figure 5d).^53^ Probe **20** is based on a D-π-A-π-D structure that exhibits intramolecular charge transfer effects. This design results in the probe exhibiting strong fluorescence in low-polarity environments (such as in LDs), while exhibiting reduced fluorescence in high-polarity environments (such as the cytoplasm). The researchers further visualized changes of LDs in a mouse model of fatty liver disease using micelles of probe **20**, successfully distinguishing between different stages of fatty liver disease. Based on these LDs-targeting probes, future designs of LDs-targeted probes could incorporate additional responsive sites, for capturing the interplay between lipid metabolism and oxidative stress, which are both central to NAFLD progression.

In addition to LDs, researchers have also visualized microenvironmental changes and variations in other active substances in different organelles for the bioimaging of NAFLD. Peroxisomes play a crucial and central role in lipid metabolism in hepatocytes, and abnormal lipid metabolism can directly affect the viscosity of peroxisomes. Therefore, visualizing the microenvironmental changes of peroxisomes can provide deep insights into the pathological mechanisms of NAFLD. As such, Li and Tang et al. developed a novel dual-modal imaging probe **21** combining NIR fluorescence and PA imaging, the probe enhances the early diagnosis of NAFLD by monitoring changes in peroxisomal viscosity, thereby offering a new perspective for investigating NAFLD (Figure 5d).^54^

Furthermore, given the rapid advancements in the integration of diagnosis and treatment, it is important to focus on the design of fluorescent probes that can be used for both the diagnosis and therapy of NAFLD. Recently, Wang, Li, Yoon and colleagues ingeniously designed a small molecule fluorescent probe **22** (Figure 5d),^55^ by integrating an HD fluorophore with an endoplasmic reticulum (ER)-targeting group (*p*-toluenesulfonamide), achieving the integration of diagnostic and therapeutic functionalities. Owing to the free rotation of the single bond in the HD structure, this probe exhibits a highly specific response to viscosity. Furthermore, ER stress is closely linked to the progression of NAFLD. Inspired by the excellent ER imaging capability of the probe, the potential of the probe for diagnosing NAFLD was evaluated (Figure 5e). The results indicated that the fluorescence intensity in NAFLD mice was significantly higher than that for normal mice, and there was a correlation between the fluorescence intensity of the probe and the severity of the lesions (Figure 5e). In addition, probe **22** was shown to be an effective type I photosensitizer, capable of causing oxidative damage to the ER of tumor cells upon prolonged irradiation. Probe **22** offers major insights for the future design of organelle-targeting multifunctional probes, which is required for the development of more effective and precise diagnostic and therapeutic tools for NAFLD.

### 3.5 Liver Fibrosis (LF)

**Figure 6:**
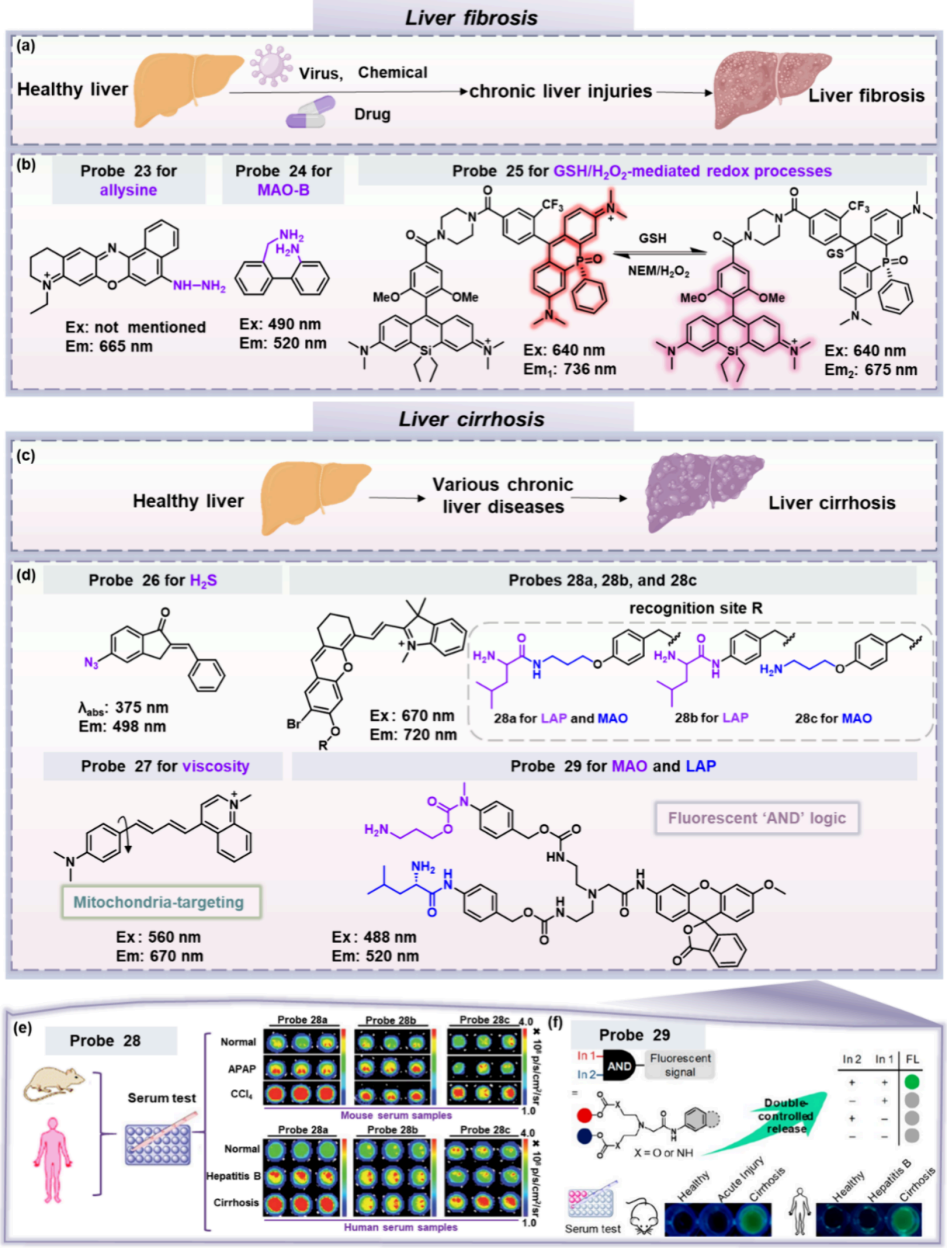
(a) LF is characterized by pathophysiological responses to chronic liver injuries, which can arise from various factors. (b) Chemical structures of fluorescent probes **23**–**25** for LF. (c) LC is seen as an irreversible stage of various chronic liver diseases. (d) Chemical structures of fluorescent probes **26**–**29** for LC. In the probe structure, the purple/blue/pink parts represent the different reactive moieties. (e) Differentiation of LC in serum can be effectively achieved through serum testing using probes **28a**–**c**. (f) Differentiation of LC by testing the blood samples from patients/mice using probe **29**. GSH, glutathione; H_2_S, hydrogen sulfide; MAO-B, monoamine oxidase B; MAO, monoamine oxidase; NEM, *N*-ethylmaleimide. Image (e) adapted with permission from ref (70). Available under a CY BY-NC 3.0 license. Copyright 2019, the authors (Y. Liu, L. Teng, C. Xu, H.-W. Liu, S. Xu, H. Guo, L. Yuan, X.-B. Zhang), published by the Royal Society of Chemistry. Image (f) adapted from ref (71). Available under a CC-BY-NC-ND 4.0 license. Copyright 2022, the authors (M. Chen, C. Wang, Z. Ding, H. Wang, Y. Wang, Z. Liu), published by American Chemical Society.

LF is characterized by pathophysiological responses to chronic liver injuries, which can arise from various factors such as viral infections, drug toxicity, and both alcoholic and nonalcoholic fatty liver diseases (Figure 6a).^56,57^ It can lead to LC and HCC, resulting in impaired liver function and potentially life-threatening consequences.^58,59^ Researchers have developed various probes to detect key biomarkers and cellular events involved in the process of LF.

Due to the overexpression of allysine during the progression of LF, Wang et al. developed a novel probe **23** (Figure 6b), using a NIR fluorophore functionalized with a hydrazine moiety.^60^ The probe exhibited a significant increase in fluorescence intensity with the addition of allysine. It enabled *in vivo* imaging of dynamic allysine changes in liver tissue at various time points during the progression of LF stimulated by the hepatotoxic substance CCl_4_. This innovative approach exhibits promise as a tool for monitoring the progression of LF and facilitating early diagnosis and treatment.

Many recent studies have indicated that serum levels of monoamine oxidase B (MAO-B) are elevated in patients with early stage LF, making it an ideal biomarker for early diagnosis. Therefore, effectively detecting changes in MAO-B levels is crucial for the prevention and diagnosis of LF. Building on this premise, Li, Tang and colleagues developed a two-photon fluorescent probe **24** by introducing MAO-B specific substrate benzylamine into 2-aminobenzeneboronic acid (Figure 6b).^61^ In a CCl_4_-induced mouse model of LF, probe **24** revealed a significant upregulation of MAO-B levels, confirming its effectiveness and sensitivity for the rapid diagnosis of early fibrosis. At the same time, researchers are actively exploring novel biomarkers to enable the early detection of LF. While various factors can induce LF, redox imbalance is considered a critical molecular mechanism in the formation of fibrotic liver tissue, leading to disturbances in GSH levels in the liver. As such, Cui et al. developed a reversible fluorescent probe **25** for imaging reversible and dynamic changes of GSH (Figure 6b).^62^ In a mouse model of LF, the probe was used to elucidate alterations in GSH levels associated with pathological processes. In mouse liver tissue, the decrease in GSH was visualized through ratiometric fluorescence imaging at various stages of CCl_4_-induced LF, confirming the sensitivity and accuracy of the probe for the early detection of LF. Overall, this work introduces an innovative approach toward LF imaging through its reversible design and NIR imaging depth. Using probe **25** as a template, future research should focus on selecting other suitable fluorophores to achieve greater separation between the two emission wavelengths, which would reduce spectral overlap and thereby enhance the accuracy of GSH detection.

### 3.6 Liver Cirrhosis (LC)

LC is seen as an irreversible stage of various chronic liver diseases (Figure 6c).^63,64^ The mechanisms underlying the development of LC involve complex biochemical processes, including persistent inflammatory responses, oxidative stress, excessive deposition of extracellular matrix, hepatocyte apoptosis, and the formation of regenerative nodules.^65,66^ Currently, research toward fluorescent probes for the diagnosis of LC primarily focuses on monitoring biomarkers that are closely associated with the pathological processes of LC (Figure 6d).

H_2_S as an active sulfur-containing compound exhibits effective antioxidant and cytoprotective effects in biological systems, and can mitigate the progression of LC to a certain extent. For H_2_S detection, Yuan et al. designed a novel probe **26** for monitoring changes in endogenous H_2_S levels in the livers of LC models (Figure 6d).^67^ To establish a reliable model of LC, mice were subjected to daily subcutaneous injections of CCl_4_ over 7 weeks to induce hepatotoxicity, liver tissues were then collected at various time points and stained with probe **26**. Utilizing two-photon imaging technology, the researchers were able to visually observe the generation and consumption of H_2_S in the LC model.

Viscosity is also linked to the advancement of LC. Based on this, Liu et al. developed a novel NIR fluorescent probe **27** (Figure 6d), which functions by monitoring changes in mitochondrial viscosity. An increase in the viscosity of cirrhotic liver tissues enabled the differentiation between normal liver tissue samples and cirrhotic liver tissue samples based on fluorescence signal changes.^68,69^

As previously discussed, dual-responsive probes exhibit improved specificity for diagnostic imaging applications compared to single-analyte fluorescent probes. Zhang et al. designed a dual-enzyme activated molecular probe **28a** and two single-enzyme activated probes **28b**, **28c** (Figure 6d).^70^ The core design of probe **28a** uses a “sequential responsive” strategy, which requires activation by two specific biomarker enzymes: LAP and monoamine oxidase (MAO), both of which are overexpressed in diseased liver states. LAP first cleaves a peptide bond in the probe, exposing an amino group, which is then oxidized by MAO and through a β-elimination reaction then releases the fluorophore. The design of this probe focuses on generating fluorescence signals through cascade reactions. Using probe **28a**, **28b**, **28c**-assisted serum imaging, the differentiation of normal, DILI and cirrhotic states was possible in mouse serum, and it was found that LAP is a key enzymatic biomarker for differentiating between normal serum and DILI serum, while MAO is a key enzymatic biomarker for differentiating between DILI serum and cirrhotic serum (Figure 6e). Moreover, the differentiation of normal, hepatitis B and LC was achieved in patients using probe-assisted serum imaging (Figure 6e). In related work, Liu et al. proposed a novel “fluorescent “AND” logic type” probe **29** (Figure 6d), which relies on the synergistic action of MAO and LAP to control the release of the fluorophore.^71^ This design highlights that the complete release of the fluorophore can only be achieved in the presence of both enzymes. Imaging experiments with this probe in living cells and mouse models confirmed its efficacy for biomedical imaging, exhibiting a strong green fluorescence signal in the serum of cirrhotic mice. Notably, this probe can successfully differentiate LC from hepatitis B using blood samples from patients (Figure 6f). Both probe **28a** and probe **29** are only activated in the presence of the two specific enzymes, effectively minimizing background signals and allowing for more accurate differentiation between LC and other liver diseases. Therefore, the application of molecular logic probes in bioimaging and disease diagnosis has been expanded, enabling valuable insights for the design of probes with high-specificity, exhibiting low-false-positives that can detect multiple biomarkers, enabling the differentiation between types of liver disease.

### 3.7 Hepatocellular Carcinoma (HCC)

**Figure 7:**
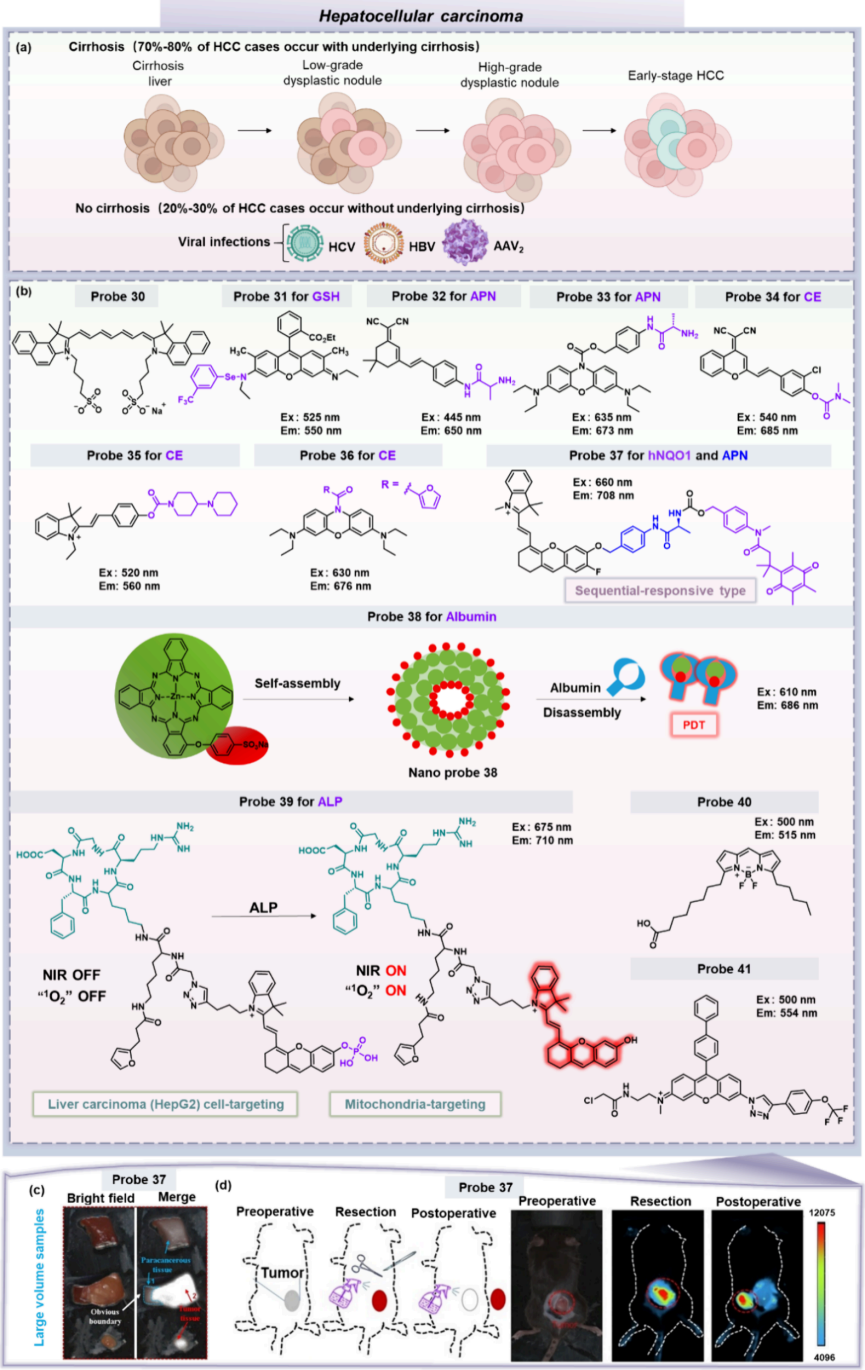
(a) Major progression and drivers in the early stages of HCC. (b) Chemical structures of fluorescent probes **30**–**39** for application in HCC. In the probe structures **30**–**39**, the purple/blue part represents the different reactive moieties; the green part of probe **39** represents the hepatocyte-targeting group. (c) Probe **37** can image tumor boundaries. (d) Probe **37** was applied to the subcutaneous tumor for performing surgical navigation. HBV, hepatitis B virus; HCV, hepatitis C virus; AAV_2_, adeno-associated virus type 2; APN, aminopeptidase N; hNQO1, NAD(P)H: quinone oxidoreductase-1; NIR, near-infrared; ^1^O_2_, singlet oxygen. Images (c), (d) adapted with permission from ref (90). Copyright 2024 Wiley-VCH GmbH.

HCC is a highly malignant tumor with persistently high incidence and mortality rates.^72^ The development of HCC is a complex, multistep process influenced by a diverse array of underlying liver disease etiologies. The risk factors associated with the progression to HCC are well-established and include LC,^73^ which results from chronic liver injury characterized by inflammation and fibrosis, as well as infections with hepatitis B virus and hepatitis C virus, among other contributing factors (Figure 7a). The early stages of HCC often lack distinct symptoms, leading to difficulties in early diagnosis and timely treatment.^74^ Therefore, precise monitoring of specific biomarkers related to HCC is crucial for early detection and timely intervention. Reported HCC biomarkers include alpha-fetoprotein,^75^ carboxylesterase (CE),^76^*etc*. In addition, serum indicators such as alanine aminotransferase, aspartate aminotransferase, and alkaline phosphatase (ALP) can point to the progression of HCC.^77^ To date, researchers have developed various fluorescent probes for monitoring HCC related-biomarkers, such as ROS,^78^ biothiols,^79^ the microenvironment^80^ and specific enzymes^77,79^ (Figure 7b).

ICG is an US Food and Drug Administration (FDA) and European Medicines Agency (EMA)-approved NIR fluorescent dye, it can be rapidly taken up by hepatocytes and accumulates in HCC tissues.^81^ Since 2008, the integration of ICG with fluorescence imaging technology has not only enhanced the detection rate of HCC, but also improved the accuracy of surgical liver resections. However, this approach continues to face challenges related to limited tissue penetration depth. Using ICG (probe **30**) in conjunction with a novel multispectral imaging technology, Gambhir, Cheng, Tian and colleagues have made significant advances required for effective surgical navigation of HCC.^82^ This imaging technology can operate in both the visible light spectrum and the NIR-I/II windows. In clinical trials involving 23 liver cancer patients, researchers found that imaging in the NIR-II window provided clearer tumor visualization compared to the NIR-I window, achieving more accurate surgical guidance. Notably, for a typical patient with extrahepatic metastasis, using the combination of probe and NIR-I/II fluorescence imaging, the authors successfully detected extrahepatic metastatic lesions that had not been identified through preoperative imaging (CT and PET), which greatly improved the accuracy of patient staging and management. Furthermore, compared to the passive targeting agent ICG, activatable probes have garnered significant attention due to their potential for higher specificity toward tumor targets. To further enhance the effectiveness of HCC detection, researchers have increasingly focused on developing biomarker-responsive fluorescent probes for accurately identifying HCC.

For biothiols, Tang et al. developed the rhodamine compound **31** containing a selenium–nitrogen (Se–N) bond, which can be cleaved by GSH, resulting in the release of the rhodamine 6G dye.^83^ Subsequently researchers employed this probe for imaging in human normal liver cell line (HL-7702) and human liver cancer cell line (HepG2). Results indicated that HL-7702 cells exhibited a strong fluorescence signal, whereas HepG2 cells displayed a significantly weaker signal. Building on this work, the Se–N motif has been used in the design of probes for RSS,^84^ laying a foundation for the development of functional probes.

Enzyme-responsive fluorescent probes have been specifically designed for the diagnosis of HCC, which enhance tumor recognition by selectively responding to overexpressed enzymes in cancer cells, such as CE, LAP, aminopeptidase N (APN) and ALP. As such, Yoon, Peng and colleagues developed a fluorescent probe **32** for detecting APN.^85^ Probe **32** comprises two distinct components: a 2-(3,5,5-trimethyl-2-cyclohexen-1-ylidene) propanedinitrile-based fluorophore and an l-alanine moiety serving as the recognition site that specifically interacts with APN. Through this APN-activated fluorescence mechanism, probe **32** achieves the highly sensitive and selective imaging of liver cancer tissues, and can monitor metastatic cancer by tracking APN activity. Subsequently, Shi, Ma and colleagues introduced a fluorescent probe **33** based on oxazine, that also used l-alanine as a recognition group for detecting APN (Figure 7b).^86^ In a HepG2 tumor-bearing mouse model, strong fluorescence induced by APN was observed. These systems indicate that a selective response by probes to APN can facilitate effective diagnosis of HCC.

For CE, Yu, Yoon and colleagues realized that rivastigmine and physostigmine, both containing carbamate moieties, can inhibit the activity of butyrylcholinesterase and acetylcholinesterase. As such they developed a highly selective fluorescent probe **34** based on the carbamate unit for the detection of CE.^87^ This design effectively avoids potential interference from acetylcholinesterase and butyrylcholinesterase, thereby enhancing imaging selectivity. Both *in vitro* and *in vivo* experiments illustrated the high sensitivity and specific response of this probe to CE activity, providing a valuable tool for the diagnosis of HCC. Similarly, Li, Ma and colleagues reported another CE-responsive fluorescent probe **35** that incorporates a bipiperidinyl into a merocyanine fluorophore.^88^ Although this probe exhibited high selectivity for CE and was able to avoid interference from other esterases, the evaluation time for probes **34** and **35** toward CE is 5 h, highlighting the significant challenge in enabling rapid fluorescence activation in response to CE. Subsequently, Yin et al. noticed that CE possesses the ability to hydrolyze compounds containing amides, as such they developed a CE-responsive fluorescent probe **36** based on an “enzymatic substrate-hydrolysis reaction” approach (Figure 7b).^89^ In simple terms, the authors synthesized a series of probes with different R substituents on the amide carbonyl, among which probe **36** enabled the rapid and highly selective imaging of CE, with a significant feature being the rapid response (reaching maximum fluorescence within just 150 s). Such a rapid response is crucial for real-time monitoring of the progression of HCC and the evaluation of therapeutic efficacy.

Dual biomarker-responsive fluorescent probes can significantly enhance the signal-to-noise ratio in imaging, providing a more reliable basis for the early diagnosis of HCC. For example, Liu, Yuan and colleagues reported a “sequential responsive” probe **37** (Figure 7b),^90^ which involves a HD modified with recognition sites specific for detecting APN, and NAD(P)H: quinone oxidoreductase-1. This design allows the probe to emit a fluorescent signal only in the presence of both biomarkers, facilitating precise imaging of tumor boundaries. Subsequently, the probe was applied to subcutaneous tumors, allowing researchers to perform surgical navigation based on the clearly visible fluorescence signals (Figure 7c, d). This work represents a novel approach for designing highly specific and accurate fluorescent probes integrating dual biomarkers for HCC.

In recent years, researchers have also constructed nanoprobes based on small molecule compounds for the detection of HCC. These nanoprobes leverage the response properties of small molecules to enhance the specificity and sensitivity of HCC detection.^91,92^ Moreover, researchers have also concentrated on the development of multifunctional probes for the detection of HCC. For instance, Huang, Choi, Nam, Yoon and colleagues introduced a novel multifunctional probe **38 (**Figure 7b),^93^ using zinc(II) phthalocyanine derivatives (as photosensitizers), featuring an amphiphilic chemical structure enabling the spontaneous assembly into uniform nanovesicle dispersions in aqueous solution. When albumin binds to these nanovesicles, the molecules are captured by the albumin, leading to disassembly of the nanovesicles and the subsequent restoration of fluorescence and ROS generation. Notably, the tumor locations in HepG2 xenograft-bearing mice were clearly visualized using probe **38**. Additionally, tumor growth in mice was significantly inhibited following treatment with probe **38** and subsequent laser irradiation. Finally, Shi et al. introduced a cyanine-based mitochondria-targeting probe **39** (Figure 7b),^94^ which uses a cyclic Arg-Gly-Asp peptide to specifically target the membranes of cancer cells. In the presence of ALP, probe **39** undergoes cleavage of the phosphate group, activating the photosensitizer and enhancing NIR/PA signals. The probe was used to selectively target HepG2 cells (ALP-overexpressed cancer cells). Notably, when exposed to red light at 660 nm, the probe not only generates singlet oxygen but also initiates RNA modifications, resulting in mitochondrial damage and significant apoptosis in tumor cells. The combination of tumor and mitochondrial targeting specificity, dual imaging capabilities (NIR/PA), and activated therapeutic potential, positions this multifunctional probe as a promising tool for both the diagnosis and potential treatment of HCC. Therefore, both probe **38** and **39** leverage biomarkers (albumin or ALP) to enhance the specificity of diagnosis and therapy, using photosensitizers. As such, future research can build on these findings, exploring novel nanomaterials and photosensitizers for developing safer and more efficient theranostic systems.

Recently, Gao, Tan, Ha, Chang and colleagues achieved the precise diagnosis of HCC using two complementary imaging probes **40** and **41**.^95^ Probe **40** exhibited high selectivity for liver cancer cells, while probe **41** was selective for healthy liver cells. The authors ultimately identified SLC27A2 as the target of probe **40** by using the CRISPR activation library, which is expressed at higher levels in HepG2 cells compared to THLE-2 cells. Through thermal proteome profiling, the protein sphingomyelin phosphodiesterase 1 (SMPD1) was identified as the target of probe **41**, with higher mRNA expression of SMPD1 in THLE-2 cells than in HepG2 cells, which explains why probe **40** and probe **41** exhibit high selectivity for liver cancer cells and healthy liver cells, respectively. Notably, when a mixture of these two probes was applied to cancerous liver tissue slices, no overlap was observed for the signals from probes **40** and **41**, resulting in a clear contrast between cancerous and normal regions. The advantage of this complementary imaging approach lies in its ability to use multiple probes that emit distinct fluorescence signals, enabling accurate differentiation between cancerous and healthy tissues. This innovative imaging approach may garner significant interest among researchers in the future, thereby enhancing the feasibility of fluorescence-guided surgery.

## 4 Conclusions

Within this perspective, we provide insights into the developmental trajectory of small molecule-based fluorescent probes for the imaging and diagnosis of liver diseases. The article focuses on several representative examples. It is clear that research in this area is resulting in the evolution of probes capable of meeting the imaging and detection requirements required for each liver disease discussed.

Initially, the probes’ capabilities were limited to detecting one specific biomarker. In general, the biomarker is one that is upregulated in the specific liver disease in question. This leads to a diagnostic problem, since the upregulation of one marker alone cannot distinguish between specific liver diseases. As such current research is directed toward probes that respond to two biomarkers. Compared to single-responsive probes, dual-responsive probes can avoid false positive results and increase the accuracy of detection. Importantly, the fundamental correlation between the two biomarkers and their synergistic effect in liver disease can be evaluated to provide an effective imaging tool to better reveal the pathological mechanism of liver disease. From the evolution of single-responsive to dual-responsive probes, researchers have also considered the implications of organelles, as they undergo specific changes during certain liver diseases. For instance, in NAFLD, the number of LDs increases. Also, during HIRI, lysosomes may become dysfunctional due to oxidative stress. Additionally, researchers are focusing on the development of multifunctional probes, which require precise structural adjustments to achieve biomarker detection, organelle targeting, hepatocyte targeting, and photodynamic therapy. These probes have the potential to simultaneously facilitate disease diagnosis and treatment, providing powerful tools for the advancement of precision medicine.

**Figure 8:**
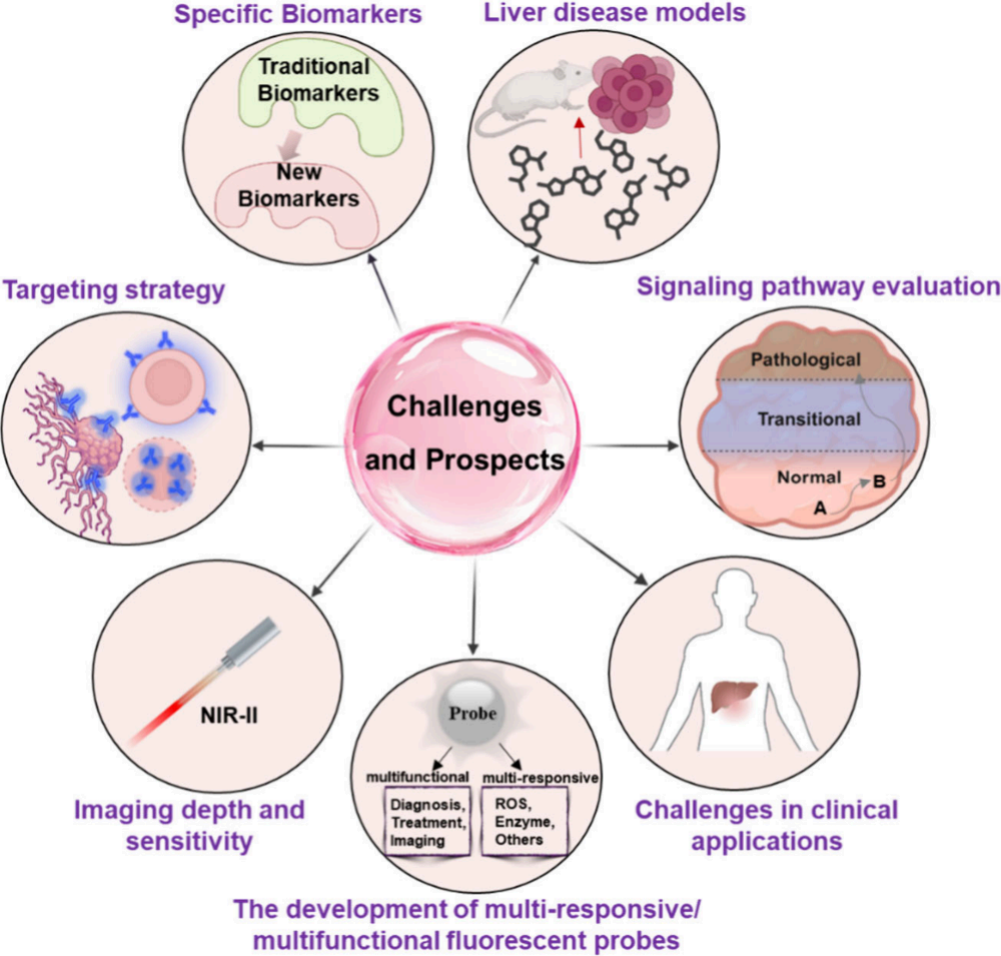
Some areas for the future development of small-molecule based fluorescent probes for detecting liver disease.

Despite notable achievements in the development of liver disease-related fluorescent probes, which have made many contributions to understanding the pathological mechanisms of liver disease, there are still many challenges before they are ready for widespread clinical application. Therefore, we recommend that researchers focus on the following areas in order to enhance the development of clinically relevant fluorescent probes for the detection of liver disease (Figure 8).

### 4.1 Specific Biomarkers

Liver disease related fluorescent probes typically focus on detecting biomarkers such as the microenvironment, active small molecules and various enzymes. However, these biomarkers are widely expressed in various liver conditions, for example, the activity of specific enzymes (such as ALP) are commonly observed in DILI and HCC, and changes in the microenvironment (such as viscosity) are commonly observed in NAFLD and HIRI. But their expression levels in different disease contexts vary significantly. Therefore, it is crucial for researchers to conduct a thorough analysis of the differential expression of these biomarkers across various liver pathologies when developing specific probes.

Additionally, there is also an urgent need to develop probes that can accurately identify biomarkers associated with specific liver diseases. The challenge with this task is to find specific biomarkers that are applicable to only one liver disease. To accomplish this, researchers must delve into the differential expression of biomarkers across different liver pathologies, with a focus on lesser-studied regulatory molecules such as proteins and nucleic acids. However, the difficulty in the development of these types of small molecule fluorescent probe means that such systems will not be available in the near future, since every time a new biomarker is proposed, a significant period of biological research is required. It is worth noting that the comprehensive analysis of multiple nonspecific biomarkers can be used as an alternative option, and this method is also an important means to improve the diagnostic accuracy for specific liver diseases. As previously stated, the progression of liver diseases entails transitions from LF to HCC, and from LC to HCC (Figure 6 and Figure 7). Consequently, there also is a pressing need to discover specific biomarkers that can effectively monitor and predict the critical junctures in these disease transitions. This endeavor is pivotal for enhancing diagnostic accuracy and predictive capabilities in the realm of liver pathology.

### 4.2 Liver Disease Models

For effective research into liver disease, we need to develop models that can accurately simulate the pathological states of human liver diseases. For instance, as mentioned in this perspective, the use of CCl_4_ as an inducer may make it impossible to generalize the research findings to a specific liver disease, since the damage it induces is not exclusive to any one type but can lead to a range of different pathological changes, such as ALI, LF, and HCC. Therefore, researchers must seek and develop new chemical induces that can more specifically mimic the particular types of liver diseases. Such agents will enable investigators to monitor biological changes more precisely under specific liver disease conditions.

### 4.3 Signaling Pathway Evaluation

Currently, fluorescent probes have been used to monitor the abnormal changes in certain biomarkers for various liver disease models. However, the coordinated alterations of other species and signaling pathways remain unknown. Therefore, researchers need to pay attention to the upstream and downstream metabolites of relevant signaling pathways. The signaling pathways involved in liver diseases are complex and diverse, including metabolism, immune response, oxidative stress and many others. These signaling pathways and mechanisms play a key role in the onset and progression of liver disease and are potential therapeutic targets. As such, understanding these pathways could help develop new therapies to prevent and treat liver disease.

### 4.4 Targeting Strategy

Targeting the liver organ is a crucial prerequisite for studying liver diseases. At the cellular/tissue level, fluorescent probes can only help in the study of liver diseases if they target hepatocytes/tissues specifically, rather than also being highly enriched in other organs outside the liver. This requires researchers to clearly distinguish between hepatocytes/tissues and other cells/tissues, and to make the utmost effort to differentiate them. At the subcellular level, cellular organelles undergo changes during the development and progression of certain liver diseases. New probes targeting specific organelles such as the Golgi apparatus, cell membranes, nucleus, and peroxisomes are still relatively scarce, limiting our understanding of their role in the pathogenisis of liver disease. Therefore, researchers should focus on the construction of probes with enhanced targeting capabilities, which is an important direction for future clinical research in liver disease detection.

### 4.5 Imaging Depth and Sensitivity

Enhancing imaging depth and sensitivity for the diagnosis of liver disease is a critical area of research. To achieve such improvements, researchers are focusing on shifting the imaging wavelength from the visible range to the NIR region, particularly the NIR-II region. However, the development of specific NIR-II fluorescence imaging probes for liver diseases is still relatively underexplored. When designing such probes, several key considerations must be addressed: enhancing the fluorescence quantum yields of the probes is one of the key factors that greatly aids clearer and more accurate *in vivo* imaging. Additionally, the development of probes that can respond to a variety of stimuli is important for activating NIR-II fluorescence signals, which can effectively reduce background noise and enhance the specificity of imaging. Furthermore, these probes should have efficient targeting capabilities to accurately target liver cells or tissues. In order to promote the clinical application of NIR-II fluorescent imaging technology for liver disease diagnosis, continuous exploration and innovation are imperative.

### 4.6 Development of Multiresponsive/Multifunctional Fluorescent Probes

As mentioned earlier, a comprehensive analysis of multiple nonspecific biomarkers can serve as an alternative option for the diagnosis of liver diseases, and understanding the signaling pathways is also very important. Therefore, it is necessary to develop suitable multiresponsive fluorescent probes. Furthermore, multifunctional fluorescent probes facilitate the integration of diagnostic and therapeutic modalities. For example, by combining biomarkers-responsive properties with photodynamic therapy ability (e.g., probe **38** and probe **39**), tumor tissues can be precisely visualized and treated. This strategy of “integrated diagnosis and treatment” represents an important development direction for the future treatment of liver diseases. However, the design and application of multiresponsive and multifunctional probes still face several limitations. For example, there is a notable deficiency in the development of dual-responsive fluorescent probes for key analytes associated with liver diseases. Additionally, many “independent optical channel-responsive” fluorescent probes suffer from spectral overlap. Future probes could be improved using the following strategies: the first strategy should focus on the selection of fluorophores with well-separated emission spectra to ensure that after interacting with different biomarkers, the emission spectra of the two fluorophores remain clearly distinguishable, which can effectively prevent spectral overlap (e.g., probe **15**). The second strategy is the potential integration of fluorescence and chemiluminescence in a single probe. Although chemiluminescence and fluorescence are different luminescent mechanisms, their emission spectra may overlap. Therefore, when selecting a chemiluminescent system, it is important to ensure that its emission wavelength is sufficiently separated from the fluorescence emission wavelength. In general, by employing these two strategies, selecting distinct fluorophores with well separated emission spectra, or developing a suitable dual-modality probe with integrated chemiluminescence and fluorescence signals, the challenge of spectral overlap may be effectively minimized. Furthermore, the limited modification sites available on fluorescent molecules restrict the number of functional groups that can be incorporated, thereby hindering the advancement of multifunctional and multiresponsive fluorescent probes. To address these challenges, future research should focus on optimizing synthetic strategies to improve the range of groups incorporated. In addition, the combination of machine learning modeling, computational chemistry, and experimental approaches may facilitate the rapid development of multifunctional and multiresponsive fluorescent probes.

### 4.7 Challenges in Clinical Applications

Fluorescent probes are powerful bioanalytical tools, offering significant potential for the diagnosis and treatment of liver diseases. However, their clinical translation still faces several challenges, among them, targeting specificity and biological safety are the most critical issues. Effective targeting is crucial for clinical applications, as probes must selectively identify diseased tissues without adversely affecting healthy tissues. However, many current fluorescent probes rely on passive accumulation in liver tumors, which limits their ability to distinguish liver tumor tissues from other pathological conditions, such as liver inflammation. Furthermore, before clinical application, rigorous evaluation of fluorescent probes is required, including a detailed assessment of biocompatibility, toxicity, and *in vivo* distribution. Additionally, the development of optimized dosage strategies and efficient probe delivery systems is critical to ensure patient safety and therapeutic efficacy.

In summary, future research must focus on identifying specific biomarkers associated with liver diseases, developing more accurate disease models, understanding the complex signaling pathways involved, improving targeted strategies, and enhancing imaging depth and sensitivity, developing multiresponsive/multifunctional fluorescent probes and promoting clinical applications. Overcoming these challenges will require interdisciplinary collaboration, innovative approaches, and sustained effort to fully harness the potential of fluorescent probes in liver disease diagnosis.
